# Changes in fibroblast growth factor 23 levels in normophosphatemic patients with chronic kidney disease stage 3 treated with lanthanum carbonate: results of the PREFECT study, a phase 2a, double blind, randomized, placebo-controlled trial

**DOI:** 10.1186/1471-2369-15-71

**Published:** 2014-05-05

**Authors:** Pablo Ureña-Torres, Dominique Prié, Karim Keddad, Peter Preston, Paul Wilde, Hong Wan, J Brian Copley

**Affiliations:** 1 Service de Néphrologie-Dialyse, Clinique du Landy, Paris, France; 2 Service de Physiologie-Explorations Fonctionnelles, Hôpital Necker-Enfants Malades, Paris, France; 3 Shire, Boulogne-Billancourt, France; 4 Shire, Wayne, Pennsylvania, USA; 5 Shire, Basingstoke, UK

**Keywords:** CKD-MBD, FGF23, Parathyroid hormone, Phosphate binders, Phosphaturia, Lanthanum carbonate

## Abstract

**Background:**

High levels of circulating fibroblast growth factor 23 (FGF23) are associated with chronic kidney disease (CKD) progression and high mortality. In the Phosphate Reduction Evaluation of FGF23 in Early CKD Treatment (PREFECT) study, we assessed the effect of reducing intestinal phosphate absorption using lanthanum carbonate on FGF23 levels in normophosphatemic patients with CKD stage 3.

**Methods:**

Thirty-five individuals were randomized to lanthanum carbonate 3000 mg/day (*n* = 23) or placebo (*n* = 12) for 12 weeks. Levels of intact FGF23 (iFGF23), C-terminal FGF23, serum and urinary phosphate and calcium, intact parathyroid hormone and 1,25-dihydroxyvitamin D were assessed.

**Results:**

The median age was 65 years in the lanthanum group and 73 years in the placebo group; 58.8% and 41.7% were men, respectively. No significant difference was seen in mean iFGF23 between groups at week 12. There was, however, a transient reduction from baseline in iFGF23 in the lanthanum group at week 1, from 70.5 pg/ml to 51.9 pg/ml, which was not seen in the placebo group; this between-group difference in percentage change from baseline was significant in *post hoc* analyses (p = 0.0102). Urinary phosphate decreased after 1 week of lanthanum treatment and remained low at week 12.

**Conclusions:**

Reducing intestinal phosphate absorption with lanthanum carbonate did not lead to sustained reductions in iFGF23 in patients with CKD stage 3, although phosphaturia decreased. This suggests that factors other than phosphate burden may be responsible for driving increases in circulating FGF23 in patients with CKD.

**Trial registration:**

ClinicalTrials.gov NCT01128179, 20 May 2010.

## Background

Fibroblast growth factor 23 (FGF23) plays a central role in mineral and bone disorders associated with chronic kidney disease (CKD-MBD) [1]. FGF23 levels are elevated early in CKD and increase as the disease progresses [2]. Increased circulating FGF23 levels are associated with an increased risk of peripheral vascular calcification [3], heart disease [4-9] and death [10-12]. In animal models, FGF23 can directly induce left ventricular hypertrophy, which can be prevented by blocking the intracellular signaling pathway stimulated by FGF23 [13]. It is not clear, however, whether increased circulating FGF23 levels in patients with CKD are causative of cardiovascular disease, or whether FGF23 is simply a biomarker for CKD-MBD severity [14].

The increase in FGF23 levels with progressing CKD probably occurs as part of the skeletal response to increased phosphate burden that results from a reduced number of functional nephrons [15]. In support of this, two studies have shown an association between increased dietary phosphate intake and serum FGF23 levels [5,16]. In a separate study, however, modest increases (33%) in dietary phosphate were not associated with increased FGF23 levels [17].

Reducing circulating FGF23 levels is a therapeutic goal for CKD-MBD, and strategies for achieving this have involved decreasing the phosphate load [18-20]. For instance, in one study of patients with CKD, a very-low-protein diet containing 0.3 g protein/kg of body weight/day (< 500 mg/day of phosphate) led to a significant decrease in FGF23 levels [21]; however, submitting individuals with CKD to such a protein-restricted diet exposes them to a high risk of malnutrition [22]. Thus, maintaining an appropriate protein and caloric intake in patients with CKD indisputably requires the decrease of intestinal absorption of phosphate [22,23].

Results of previous studies are conflicting, showing that treatment with intestinal phosphate binders can lead to no evidence of change, reductions or increases in FGF23 levels [18,19,24]. The primary objective of the PREFECT study was to assess the effects of 12 weeks of treatment with lanthanum carbonate compared with placebo on intact FGF23 (iFGF23) levels in normophosphatemic patients with CKD stage 3.

Secondary and tertiary objectives were to assess the effects of lanthanum carbonate on other factors that are altered in CKD-MBD, including circulating levels of intact parathyroid hormone (iPTH), 1,25-dihydroxyvitamin D, calcium, and phosphate.

## Methods

### Study population

Men and non-pregnant, non-lactating women aged 18 years or over with CKD stage 3 were included in the study. Patients had to have been under the care of a physician for at least 2 months, and not be expected to begin dialysis for at least 6 months. At screening, patients had to have serum phosphate levels within the normal range (0.808–1.55 mmol/l), an estimated glomerular filtration rate (eGFR) (estimated using the Cockcroft–Gault and Modification of Diet in Renal Disease [MDRD] formulae [25]) of 30–59 ml/min/1.73 m^2^, C-terminal FGF23 (cFGF23) levels ≥ 50.0 RU/ml and 25-hydroxyvitamin D levels ≥ 20 ng/ml. Patients also had to have an adequate protein diet that included two or three portions of protein-rich food per day, as determined at screening. Those who required vitamin D supplements or compounds containing calcium, phosphate, aluminum or magnesium were excluded. Individuals were also excluded if they had: acute kidney failure; rapidly progressing glomerulonephritis; cirrhosis or other clinically significant liver disease; human immunodeficiency virus infection; life-threatening malignancy or multiple myeloma; or any clinically significant illness that would, in the opinion of the investigator, impair their ability to give informed consent or to take part in or complete the study. Patients with a history of gastrointestinal disorders, alcohol or substance abuse, or poor compliance (< 60% or > 120% tablets taken) were excluded, as were vegetarians and patients allergic to iodine.

Patients were withdrawn from the study if: their serum phosphate concentration fell below 0.60 mmol/l; their medical condition deteriorated significantly in the opinion of the investigator; they had a major change in diet; they commenced dialysis or required a kidney transplant; they had unmanageable adverse events; or if they became pregnant.

The study was conducted in accordance with all applicable regulations, the International Conference on Harmonisation guideline for Good Clinical Practice [26], and the Declaration of Helsinki and its subsequent revisions [27]. The study protocol and informed consent documents received ethical approval from the institutional ethical review committee: Comité de Protection des Personnes, Ile de France II. Necker Hospital, Paris, France. All patients provided written informed consent before participating in the study.

### Study design

**Figure 1:**
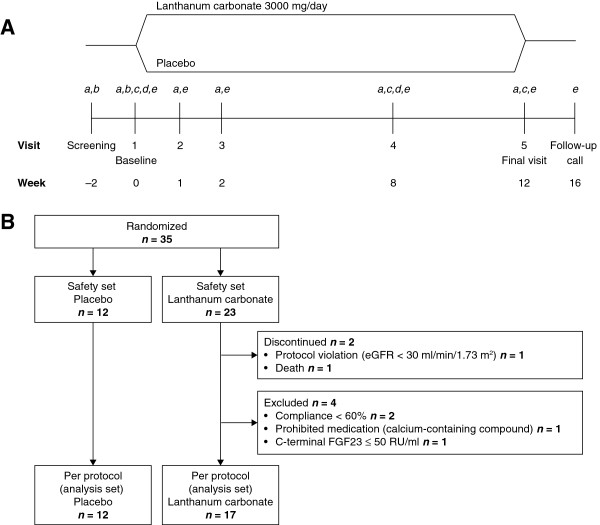
**(A) Study design and (B) population.** Schedule of assessments: *a* = iFGF23, cFGF23, 25-hydroxyvitamin D, 1–25 dihydroxyvitamin D, bone-specific ALP, iPTH; *b* = eGFR; *c* = 24-h urinary phosphate, calcium creatinine and urea nitrogen; *d* = iGFR, ionized calcium, CrossLaps^®^; *e* = adverse events. ALP, alkaline phosphatase; cFGF23, C-terminal fibroblast growth factor 23; eGFR, estimated glomerular filtration rate; iFGF23, intact fibroblast growth factor 23; iGFR, iohexol glomerular filtration rate; iPTH, intact parathyroid hormone.

This was a phase 2a, double-blind, placebo-controlled, proof-of-concept study carried out between November 2010 and May 2012 (ClinicalTrials.gov identifier: NCT01128179). Participants were screened within a 2-week period, then returned for a baseline visit at which their eligibility for inclusion in the study was confirmed before they were randomized, by sequential allocation of a unique 2-digit number by the investigator that matched a 2-digit number on the study medication. Treatment was assigned by a randomization schedule and patients received either lanthanum carbonate (FOSRENOL^®^, Shire, Nyon, Switzerland) 1000 mg three times daily, or a placebo equivalent for 12 weeks (Figure 1A), details of which were in the form of code-break envelopes held at the investigational site. The investigational product was to be chewed rather than swallowed whole, and was recommended to be taken with meals. Patients returned to the clinic site for visits 1, 2, 8 and 12 weeks after the baseline visit, and a follow-up call was scheduled for week 16, or 30 days after the end of treatment if the investigational product was discontinued before study completion.

The primary endpoint was iFGF23 level at week 12. Secondary endpoints were: iPTH, 1,25-dihydroxyvitamin D, phosphate, total calcium and calcium × phosphate product; 24-h urinary phosphate and calcium excretion; urinary creatinine and urea nitrogen concentrations. Tertiary endpoints were: 25-hydroxyvitamin D, ionized calcium, creatinine and cFGF23; GFR as measured by plasma iohexol clearance (iGFR) according to previously described methods [28], and eGFR; CrossLaps^®^ (a measure of bone resorption; IDS Plc, UK); and bone-specific alkaline phosphatase.

Blood samples were collected at screening and all subsequent visits for measurement of iFGF23, cFGF23, 25-hydroxyvitamin D, 1,25-dihydroxyvitamin D, bone-specific alkaline phosphatase and iPTH levels. Twenty-four-hour urine samples were collected at all visits from baseline to week 12 for assessment of phosphate, calcium, creatinine and urea nitrogen levels. One day prior to the visit, each patient was asked to discard their first morning urination, to note the time on the urinary bottle, and then to collect the urine of all subsequent micturitions for 24 h. The time of the last urination was also noted on the bottle. eGFR was assessed at screening, baseline and week 12. iGFR, ionized calcium and CrossLaps^®^ were assessed at baseline and week 12. Vital signs and adverse events were monitored at every visit from screening to the final visit. Adverse events were also recorded on the follow-up call. Compliance was calculated based on the number of tablets remaining in medicine bottles returned at the end of the study.

The study was conducted at a single center (Service de Néphrologie-Dialyse, Clinique du Landy, Paris, France), and measurement of GFR and biochemical evaluations were performed at a single department (Service de Physiologie-Explorations Fonctionnelles, Hôpital Necker-Enfants Malades, Paris, France).

### Statistical analyses

Previous work by one of the authors (DP) showed fasting FGF23 levels in patients with CKD to be log-normally distributed with a coefficient of variation of the logged data of 1.13 (author’s unpublished data). A sample size of 33 participants randomized in a 2:1 ratio (22 receiving lanthanum carbonate and 11 receiving placebo) was estimated to be sufficient to detect a ratio of 1.15 in the logged means, or a 40% reduction in mean iFGF23 levels in the lanthanum carbonate group compared with the placebo group at 12 weeks. This assumed 80% power and a two-sided significance level of 5%.

Patients who were randomized and received at least one dose of the investigational product were included in the safety analysis set. Patients in the safety analysis set who had at least one post-dose assessment were included in the full analysis set. The per protocol set consisted of patients who had at least one dose of investigational product in addition to primary data assessment available from week 2 or later, and who did not have any pre-defined protocol deviations that might affect the primary variable. The per protocol set was the primary analysis population for this proof-of-concept study.

Data were presented as descriptive statistics, which are shown as mean ± standard deviation (SD) or percentage change from baseline in the least-squares (LS) mean ± standard error. Statistical analyses were performed using SAS^®^ version 9.1 (SAS Institute, Cary, NC, USA). Differences in the primary endpoint, iFGF23 levels, between the placebo and lanthanum carbonate groups at week 12 were evaluated using analysis of covariance (ANCOVA) and log-transformed values, with baseline iFGF23 as a covariate. ANCOVA models were also used to evaluate the statistical significance of differences in secondary and tertiary variables using change from baseline to week 12, with treatment group as a factor and the corresponding value at baseline as a covariate. Missing data were accounted for using the last observation carried forward method. In *post hoc* analyses, ANCOVA was used to evaluate the statistical difference in the percentage change from baseline in the LS means between the lanthanum carbonate and placebo groups by week.

## Results

### Study population

In total, 51 patients were screened, of whom 35 met the inclusion criteria and entered the study. These individuals made up the safety analysis set and were randomized such that 23 patients received lanthanum carbonate and 12 received placebo (Figure 1B). All patients included in the safety analysis set also made up the full analysis set. In the lanthanum carbonate group, two participants did not complete the study: one died (not considered to be treatment related); the other was withdrawn because of a protocol violation (eGFR < 30 ml/min/1.73 m^2^). Also in the lanthanum carbonate group, four patients completed the study but met exclusion criteria and were not included in the per protocol analysis set.

Baseline characteristics of the per protocol analysis set were similar to the safety analysis set (Table 1); however, there were some numerical differences between the lanthanum carbonate and placebo groups. The median age was 65 years (range: 38–84 years) in the lanthanum carbonate group and 73 years (range: 43–87 years) in the placebo group. In the lanthanum carbonate group, 58.8% of patients were men, compared with 41.7% of those in the placebo group. Most participants were white (lanthanum carbonate group: 70.6%; placebo group: 75.0%), and there was a higher proportion of black patients in the lanthanum carbonate group (29.4%) than in the placebo group (16.7%). Individuals in the lanthanum carbonate group had a lower iGFR (mean: 42.2 ml/min/1.73 m^2^, standard deviation [SD]: 10.1 ml/min/1.73 m^2^) than the placebo group (mean: 48.0 ml/min/1.73 m^2^, SD: 13.1 ml/min/1.73 m^2^) at baseline. Mean treatment compliance, measured by the proportion of tablets left in the medicine bottle at the treatment visit, was 87.5% (SD: 20.1%) in the lanthanum carbonate group and 91.0% (SD: 13.0%) in the placebo group.

**Table 1:** Patient demographics at baseline (week 0, safety analysis set and per protocol analysis set)

	**Safety set**		**Per protocol set**	
	**Lanthanum carbonate**	**Placebo**	**Lanthanum carbonate**	**Placebo**
	**(*n*** **= 23)**	**(*n*** **= 12)**	**(*n*** **= 17)**	**(*n*** **= 12)**
Age, years				
Mean (SD)	66.0 (13.9)	69.4 (13.2)	65.6 (14.7)	69.4 (13.2)
Median	65.0	72.5	65	73
Range	38–84	43–87	38–84	43–87
Gender, *n* (%)				
Male	13 (56.5)	5 (41.7)	10 (58.8)	5 (41.7)
Ethnicity, *n* (%)				
White	16 (69.6)	9 (75)	12 (70.6)	9 (75.0)
Black	6 (26.1)	2 (16.7)	5 (29.4)	2 (16.7)
Asian	0 (0.0)	1 (8.3)	0 (0.0)	1 (8.3)
Body mass index, kg/m^2^, mean (SD)	29.9 (5.1)	30.1 (5.1)	29.4 (4.9)	30.1 (5.1)
iGFR (ml/min/1.73 m^2^), mean (SD)	42.5 (10.7)	48.0 (13.1)	42.2 (10.1)	48.0 (13.1)

iGFR, iohexol glomerular filtration rate; SD, standard deviation.

### iFGF23 and cFGF23 levels

**Figure 2:**
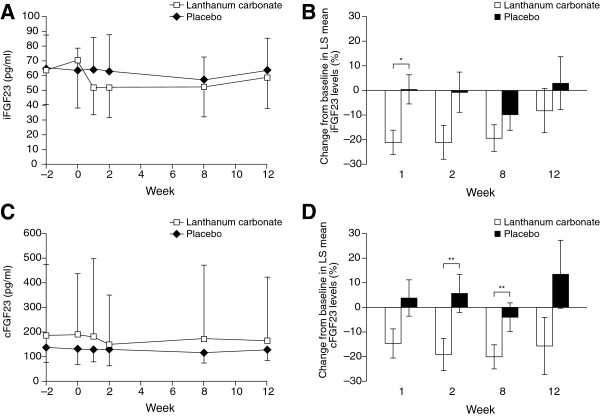
**iFGF23 and cFGF23 levels over time. (A)** Mean iFGF23 levels, **(B)** LS mean percentage change from baseline in iFGF23 levels, **(C)** mean cFGF23 levels and **(D)** LS mean percentage change from baseline in cFGF23 levels. *p = 0.0102; **p < 0.05 vs placebo. Statistical significance was assessed using analysis of covariance. Lanthanum carbonate, *n* = 17; placebo *n* = 12. For cFGF23 (c and d) one patient was missing from the placebo group at week 1. **(A)** and **(C)** show mean values ± standard deviation; **(B)** and **(D)** show LS mean ± standard error. cFGF23, C-terminal fibroblast growth factor 23; iFGF23, intact fibroblast growth factor 23; LS, least-squares.

Numerical changes in mean iFGF23 levels were seen between the lanthanum carbonate and placebo groups throughout the treatment period, although there was no statistically significant difference in the per protocol set between the groups at week 12, the primary endpoint of the study (p = 0.3186 from F-test of logged LS mean iFGF23 values) (Figure 2A). This was also true of the safety/full analysis set, in which there was no statistically significant difference in LS mean iFGF23 at week 12 (p = 0.6330). Across all the analyses, the results were similar for the per protocol set and the safety analysis set, and these groups had similar baseline characteristics; therefore, only efficacy results for the per protocol analysis set are presented.

In the lanthanum carbonate group, mean iFGF23 levels decreased from 70.5 pg/ml (SD: 32.2 pg/ml) at baseline to 51.9 pg/ml (SD: 18.2 pg/ml) at week 1; however, they subsequently increased to 58.8 pg/ml (SD: 20.6 pg/ml) by week 12. For patients given placebo, there was negligible change in mean iFGF23 levels throughout the study (baseline: 63.7 pg/ml (SD: 14.8 pg/ml); week 12: 63.7 pg/ml (SD: 21.7 pg/ml).

The percentage change from baseline in LS mean iFGF23 levels was also retrospectively assessed for each treatment visit (Figure 2B). In this *post hoc* analysis, the reduction in iFGF23 in the lanthanum carbonate group compared with the placebo group was statistically significant at week 1 (p = 0.0102); however, a statistically significant difference was not seen at subsequent time points.

Mean FGF23 levels were also assessed using an assay to detect the tertiary variable, cFGF23 (Figure 2C). *Post hoc* analysis showed that there was a reduction at all weeks in the percentage change from baseline in LS mean cFGF23 levels in the lanthanum carbonate group, whereas these levels increased or were slightly reduced (week 8) in the placebo group (Figure 2D). The between-group difference was significant 2 and 8 weeks after the start of treatment (p < 0.05, ANCOVA), but not after 1 or 12 weeks.

### Serum and urinary phosphate and calcium levels

**Figure 3:**
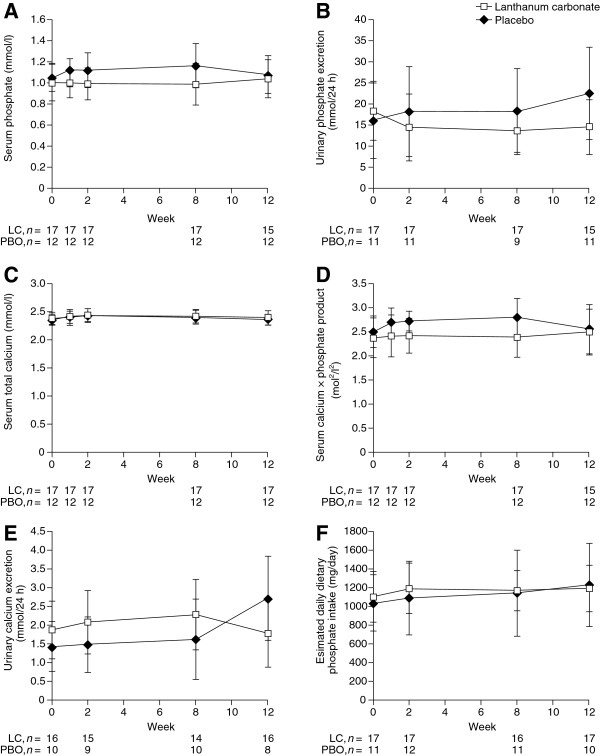
**Changes in phosphate and calcium levels throughout the study. (A)** Serum phosphate, **(B)** 24-h urinary phosphate excretion, **(C)** serum total calcium, **(D)** serum calcium × phosphate product, **(E)** 24-h urinary calcium excretion and **(F)** estimated daily dietary phosphate intake. Graphs show mean values ± standard deviation. LC, lanthanum carbonate; PBO, placebo.

Secondary variables, serum and urinary phosphate and calcium levels were assessed during the study and between-group differences were analyzed statistically at week 12 (Figure 3; Additional file 1: Table S1). Serum phosphate was lower in the lanthanum carbonate group than in the placebo group throughout the study, including the baseline visit, but there was no significant difference in serum phosphate between groups after 12 weeks (Figure 3A). Twenty-four-hour urinary phosphate excretion was generally higher in the placebo group than in the lanthanum carbonate group, and this difference was significant at week 12 (p = 0.0162) (Figure 3B, Additional file 1: Table S1). In the lanthanum carbonate group, phosphate excretion decreased from 18.2 mmol/24 h (SD: 6.8 mmol/24 h) at baseline to 14.4 mmol/24 h (SD: 7.9 mmol/24 h) at week 2 and remained at this level until week 12 (14.5 mmol/24 h [SD: 6.4 mmol/24 h]). Phosphate excretion in the placebo group increased from a mean of 16.2 mmol/24 h (SD: 9.1 mmol/24 h) at baseline to 22.5 mmol/24 h (SD: 10.9 mmol/24 h) by week 12.

Serum total calcium level and calcium × phosphate product were similar in the placebo and lanthanum carbonate groups, and remained constant throughout the study (Figure 3C and 3D). Urinary calcium levels were generally higher in the lanthanum carbonate group than in the placebo group (Figure 3E); however, by week 12, urinary calcium levels had increased in the placebo group (2.72 mmol/24 h [SD: 1.12 mmol/24 h]) and had fallen to below placebo levels in the lanthanum carbonate group (1.78 mmol/24 h [SD: 0.90 mmol/24 h]). There was a significant difference between the decrease in urinary calcium from baseline in the lanthanum carbonate group and the increase from baseline in the placebo group at week 12 (p = 0.0371; Additional file 1: Table S1).

Daily dietary phosphate intake was not measured directly in this study, but was estimated retrospectively using urinary urea nitrogen measurements. Estimated daily phosphate intake was similar both between the two treatment groups, and within each group throughout the study (Figure 3F).

### Intact parathyroid hormone and 1,25-dihydroxyvitamin D levels

Mean iPTH and 1,25-dihydroxyvitamin D levels for each treatment group were measured as secondary variables throughout the study (Additional file 2: Figure S1). Mean iPTH levels were similar in the placebo and lanthanum carbonate groups: there was a decrease from baseline (placebo: 75.8 pg/ml [SD: 43.9 pg/ml], lanthanum carbonate: 69.4 pg/ml [SD: 30.4 pg/ml]) to week 1 (placebo: 62.2 pg/ml [SD: 38.1 pg/ml], lanthanum carbonate: 51.6 pg/ml [SD: 22.2 pg/ml]) in both groups, but levels returned to close to baseline values by week 12. In patients who received placebo, there was a small numerical decrease in mean 1,25-dihydroxyvitamin D levels by week 12 compared with baseline (-9.5 pg/ml [SD: 19.6]), whereas there was no change from baseline to week 12 in the lanthanum carbonate group (0.1 pg/ml [SD: 12.2 pg/ml]). No significant difference in the change from baseline was seen between the LS means of the lanthanum carbonate and placebo groups for iPTH (p = 0.2995) or for 1,25-dihydroxyvitamin D (p = 0.3252; Additional file 1: Table S1).

Serum or urinary levels of other relevant variables (urinary fractional phosphate excretion, creatinine, urea nitrogen, 25-hydroxyvitamin D, ionized calcium, CrossLaps^®^, iGFR and bone-specific alkaline phosphatase) are shown in Additional file 1: Table S1. No significant between-group differences were seen between weeks 0 and 12 for these factors.

### Post hoc subgroup analysis of iFGF23 levels

Retrospective subgroup analyses were undertaken to assess whether baseline differences between the lanthanum carbonate and placebo groups affected circulating iFGF23 levels over time (Additional file 3: Figure S2). Patients with baseline eGFR below 45 ml/min (CKD stage 3b) showed greater reductions in iFGF23 in both the lanthanum carbonate and placebo groups, than individuals with a baseline eGFR in the range 45–60 ml/min; however, no significant difference was seen between placebo and lanthanum carbonate treatment in either eGFR group. Covariate analyses found no association of the percentage change from baseline of iFGF23 at any time point with baseline cFGF23, baseline iPTH, estimated daily phosphate intake or patient gender.

### Safety evaluation

A summary of adverse events experienced in the study is given in Table 2. In total, 30.4% of patients experienced adverse events in the lanthanum carbonate group compared with 16.7% in the placebo group. The most frequently observed adverse events were gastrointestinal (17.4% for lanthanum carbonate vs 16.7% for placebo), including constipation, dry mouth and acute pancreatitis. Two patients (8.7%) treated with lanthanum carbonate experienced serious adverse events but neither event was considered to be related to the investigational product: one patient experienced acute pancreatitis; the other died following cardiac failure and myocardial ischemia.

**Table 2:** Summary of adverse events in the safety analysis set

	**Lanthanum carbonate (*n*** **= 23)**			**Placebo (*n*** **= 12)**		
	***n***	**(%)**	**Events**	***n***	**(%)**	**Events**
Any adverse event	7	(30.4)	15	2	(16.7)	3
Related to investigational product	2	(8.7)	6	1	(8.3)	1
Moderate or severe adverse event	2	(8.7)	4	–	–	–
Serious adverse event	2	(8.7)	4	–	–	–
Related to investigational product	–	–	–	–	–	–
Leading to withdrawal of investigational product	1	(4.3)	1	–	–	–
**Type of adverse event**						
Gastrointestinal disorders	4	(17.4)	4	2	(16.7)	3
Abdominal discomfort	–	–	–	1	(8.3)	1
Constipation	2	(8.7)	2	–	–	–
Diarrhea	–	–	–	1	(8.3)	1
Dry mouth	1	(4.3)	1	–	–	–
Acute pancreatitis	1	(4.3)	1	–	–	–
Vomiting	–	–	–	1	(8.3)	1
Cardiac disorders	1	(4.3)	3	–	–	–
Cardiac failure	1	(4.3)	2	–	–	–
Myocardial infarction	1	(4.3)	1	–	–	–
Other organ system classes						
Thirst	1	(4.3)	1	–	–	–
Bronchitis	1	(4.3)	1	–	–	–
Anorexia	1	(4.3)	1	–	–	–
Headache	1	(4.3)	1	–	–	–
Polyuria	1	(4.3)	1	–	–	–
Erythema	1	(4.3)	1	–	–	–
Hyperhidrosis	1	(4.3)	1	–	–	–
Hypertension	1	(4.3)	1	–	–	–

## Discussion

In this study, the effect of lanthanum carbonate on circulating FGF23 levels and other biochemical markers of CKD-MBD was observed over a 12-week period. The primary objective of the study was not met, in that no statistically significant difference was seen in circulating iFGF23 levels between patients treated with placebo and those receiving lanthanum carbonate after 12 weeks of treatment.

It was expected that lanthanum carbonate treatment would reduce phosphate load, observed as reductions in serum phosphate concentration and urinary phosphate excretion, and subsequently that circulating FGF23 levels would be reduced. This hypothesis was supported by a previous study of 18 patients with CKD stage 3, which showed that FGF23 levels were significantly reduced after 4 weeks of treatment with lanthanum carbonate 2250 mg/day, and that urinary phosphate excretion was reduced accordingly [19]. In the present study in normophosphatemic individuals with CKD, however, the trend in urinary phosphate levels did not correspond to that of either circulating iFGF23 or cFGF23 levels. Notably, after an initial reduction in urinary phosphate excretion following drug administration, urinary phosphate levels tended to remain constant in the lanthanum carbonate group, whereas circulating iFGF23 levels started to rise again from week 2. Although a correlation between these factors was not specifically assessed, the differing trends between urinary phosphate excretion and circulating iFGF23 suggest that phosphate burden may not be the main factor driving the increase in FGF23 levels seen in CKD-MBD. This supports the suggestion that FGF23 could be a useful biomarker for detecting CKD-MBD progression in advance of observed changes in serum phosphate levels [29].

One possibility is that reductions in intestinal phosphate absorption and in extracellular phosphate burden in the first 2 weeks of treatment in the present study resulted in reduced FGF23, but that underlying bone disease and altered osteocyte/osteoblast/osteoclast function or a compensatory mechanism drove increases in FGF23 production or decreases in FGF23 degradation after this time. This is consistent with an idea recently put forward that there are two distinct components of the FGF23 regulatory mechanism: one that is affected by changes in circulating phosphate concentrations, and another that is linked to the progression of CKD [20].

Although covariate analyses showed no statistically significant influence of baseline parameters on iFGF23 levels between the treatment groups at baseline, there were some potentially clinically significant differences between the groups. First, whereas patients in the placebo group had GFR levels corresponding to CKD stage 3a, the lanthanum carbonate group had CKD stage 3b. Secondly, more men and more black patients were included in the lanthanum carbonate group than in the placebo group; both race and gender have been reported to have a potential influence on circulating FGF23 levels [10,29,30]. Finally, there may have been imbalances in other as yet unidentified factors that contribute to FGF23 regulation.

The effect of lanthanum carbonate treatment on circulating FGF23 levels in patients with CKD has been reported in several previous studies (Table 3). Gonzalez-Parra et al. demonstrated a decrease in FGF23 in patients with CKD stage 3 after 4 weeks of treatment with lanthanum carbonate [19]. Isakova et al. saw a reduction in FGF23 values after 12 weeks of treatment with lanthanum carbonate in patients with CKD stages 3 or 4, but only if administered in combination with a phosphate-restricted diet [20]. Other studies on lanthanum carbonate in patients with CKD stages 3–4 have shown no significant change in circulating FGF23 levels using cFGF23 or iFGF23 after 2 weeks [24] or 9 months [31]. FGF23 has also been assessed following treatment with sevelamer or calcium-based phosphate binders [18,31,32]. Sevelamer treatment led to a reduction in circulating FGF23 levels in patients with CKD stages 3–4 after 6 weeks [18], and 9 months of treatment [31], and after 8 weeks in patients with CKD stage 4 [32]. There was a less consistent effect of calcium-based phosphate binders on FGF23 in these studies. The inconsistent results between these publications suggest that further studies are required, using a larger sample size and controlled dietary phosphate, to clearly determine whether there is any differing effect between intestinal phosphate binder types and FGF23. Notably, the exact stage of CKD varies between the studies discussed here and this may have a substantial effect on the FGF23 response.

**Table 3:** Comparison of published studies that assess the effect of phosphate binders on FGF23

**Reference**	**Intervention**	**iFGF23/cFGF23**	**N**	**CKD stage**	**Duration**	**Results**
Isakova et al., CJASN [20]	Placebo; LC; P-restricted diet + placebo; P-restricted diet + LC	cFGF23 only	39	3–4	12 weeks	Significant 35% reduction in FGF23 in the P-restricted + LC group at week 12 vs baseline.
						No significant change in FGF23 from baseline in other groups.
Yilmaz et al., AJKD [32]	Sevelamer; Calcium acetate	iFGF23	100	4	8 weeks	Sevelamer group: reduction (27.1%) at week 8.
						Calcium acetate group: increase (3.5%) at week 8. Significance of changes from baseline not specified.
Oliveira et al., CJASN [18]	Calcium acetate; Sevelamer hydrochloride	iFGF23	40	3–4	6 weeks	For patients with stage 3 CKD:
						• Sevelamer group: significant reduction in FGF23 at week 6 (34.6%).
						• Calcium group: non-significant reduction in FGF23 at week 6 (24.7%).
Isakova et al., NDT [24]	LC; Placebo (both on P-controlled diet either 750 mg or 1500 mg)	cFGF23	16	3–4	2 weeks	No significant change from baseline.
						Slight increase from baseline in the group ingesting 1500 mg P + placebo.
Block et al., JASN [31]	Placebo; Calcium acetate; Sevelamer; LC	cFGF23 and iFGF23	148	3–4	9 months	No significant differences in cFGF23.
						Change from baseline of iFGF23:
						• Sevelamer: small but significant reduction.
						• Calcium acetate: small but significant increase.
						• LC and Placebo: no change.
Gonzalez-Parra et al., NDT [19]	LC	cFGF23	18	3	4 weeks	Significant reduction (21.8%) from baseline of cFGF23.

LC: lanthanum carbonate, P: phosphate, FGF23: fibroblast growth factor 23; iFGF23: intact FGF23; cFGF23: carboxy-terminal FGF23.

One of the strengths of the present work is that the patient population had a measured GFR that allowed CKD stage 3 to be very well defined. A limitation of the present study was the small number of patients recruited, which could be responsible for the high within-group variability, and the lack of significance in statistical tests. The calculations used to determine the study sample size were powered to detect a 40% decrease in FGF23, whereas the greatest reduction in FGF23 that was observed was 20%: a larger sample size would be needed to detect statistically significant differences between the groups based on this effect size. A second limitation was that the completeness of 24 h urinary samples was not assessed, although urinary creatinine was assessed. Thirdly, dietary phosphate was not controlled throughout the study. Patients were encouraged not to alter their eating habits during the study and protein intake was retrospectively estimated based on urea nitrogen levels; however, non-protein sources of phosphate would not necessarily be identified using this method. This limitation is particularly important in light of a recent paper that saw an effect of lanthanum carbonate on circulating FGF23 levels only in combination with a phosphate-restrictive diet [20].

## Conclusions

Circulating iFGF23 levels were not significantly reduced in patients with CKD stage 3 after 12 weeks of treatment with lanthanum carbonate compared with placebo, despite a reduction in urinary phosphate excretion and a transient reduction in iFGF23 at week 1. This suggests that phosphate burden may not be the main driver of the increase in circulating FGF23. Further studies with a larger sample size are required to confirm whether reducing intestinal phosphate absorption by phosphate binders is sufficient to bring about long-term reductions in circulating FGF23 levels.

## Abbreviations

ANVOCA: Analysis of covariance; CKD: Chronic kidney disease; CKD-MBD: Chronic kidney disease-mineral and bone disorder; cFGF23: C terminal fibroblast growth factor 23; eGFR: Estimated glomerular filtration rate; FGF23: Fibroblast growth factor 23; iFGF23: Intact fibroblast growth factor 23; iPTH: Intact parathyroid hormone; LS: Least-squares; PREFECT: Phosphate reduction evaluation of FGF23 in early CKD Treatment; SD: Standard deviation.

## Competing interests

This study was funded by Shire Development LLC. KK, PW, HW, and JBC are employees of Shire and own stock in Shire. PP is a consultant to Shire. PU-T has received honoraria, research funds, and consulting fees from Abbott, Amgen, Fresenius, Novartis/Genzyme, Reata and Shire. DP has received honoraria from Shire. The results presented in this paper have not been published previously in whole or in part, except in abstract format.

## Authors’ contributions

PU-T and DP were involved in the study design and collection of clinical data. PP and HW carried out the statistical analysis. KK, PW and JBC participated in the study design and coordination. All authors were involved in the interpretation of the data, reviewed each draft of the manuscript and read and approved the final manuscript.

## Pre-publication history

The pre-publication history for this paper can be accessed here:

http://www.biomedcentral.com/1471-2369/15/71/prepub

## Supplementary Material

Additional file 1: Table S1 Change from baseline to week 12 in secondary and tertiary variables. Click here for file

Additional file 2: Figure S1 Change in **(a) **intact parathyroid hormone and **(b) **1,25-dihydroxyvitamin D throughout the study. Click here for file

Additional file 3: Figure S2 *Post hoc *analysis of iFGF23 levels. Graphs show LS mean percentage change from baseline of iFGF23 by treatment and by baseline levels of **(a) **estimated glomerular filtration rate (eGFR), **(b) **C-terminal FGF23 (cFGF23), **(c)** intact parathyroid hormone (iPTH) and by **(d) **estimated daily phosphate intake and **(e) **gender. Click here for file

## References

[B1] Berndt TJ Schiavi S Kumar R “Phosphatonins” and the regulation of phosphorus homeostasis Am J Physiol Renal Physiol 2005 289 F1170 F1182 10.1152/ajprenal.00072.2005 16275744

[B2] Fliser D Kollerits B Neyer U Ankerst DP Lhotta K Lingenhel A Ritz E Kronenberg F Kuen E Konig P Kraatz G Mann JF Muller GA Kohler H Riegler P Fibroblast growth factor 23 (FGF23) predicts progression of chronic kidney disease: the Mild to Moderate Kidney Disease (MMKD) Study J Am Soc Nephrol 2007 18 2600 2608 10.1681/ASN.2006080936 17656479

[B3] Inaba M Okuno S Imanishi Y Yamada S Shioi A Yamakawa T Ishimura E Nishizawa Y Role of fibroblast growth factor-23 in peripheral vascular calcification in non-diabetic and diabetic hemodialysis patients Osteoporos Int 2006 17 1506 1513 10.1007/s00198-006-0154-6 16896512

[B4] Gutierrez OM Januzzi JL Isakova T Laliberte K Smith K Collerone G Sarwar A Hoffmann U Coglianese E Christenson R Wang TJ DeFilippi C Wolf M Fibroblast growth factor 23 and left ventricular hypertrophy in chronic kidney disease Circulation 2009 119 2545 2552 10.1161/CIRCULATIONAHA.108.844506 19414634 PMC2740903

[B5] Gutierrez OM Wolf M Taylor EN Fibroblast growth factor 23, cardiovascular disease risk factors, and phosphorus intake in the health professionals follow-up study Clin J Am Soc Nephrol 2011 6 2871 2878 10.2215/CJN.02740311 22034506 PMC3255372

[B6] Kendrick J Cheung AK Kaufman JS Greene T Roberts WL Smits G Chonchol M FGF-23 associates with death, cardiovascular events, and initiation of chronic dialysis J Am Soc Nephrol 2011 22 1913 1922 10.1681/ASN.2010121224 21903574 PMC3187186

[B7] Kirkpantur A Balci M Gurbuz OA Afsar B Canbakan B Akdemir R Ayli MD Serum fibroblast growth factor-23 (FGF-23) levels are independently associated with left ventricular mass and myocardial performance index in maintenance haemodialysis patients Nephrol Dial Transplant 2011 26 1346 1354 10.1093/ndt/gfq539 20813767

[B8] Ix JH Katz R Kestenbaum BR de Boer IH Chonchol M Mukamal KJ Rifkin D Siscovick DS Sarnak MJ Shlipak MG Fibroblast growth factor-23 and death, heart failure, and cardiovascular events in community-living individuals: CHS (Cardiovascular Health Study) J Am Coll Cardiol 2012 60 200 207 10.1016/j.jacc.2012.03.040 22703926 PMC3396791

[B9] Peiskerova M Kalousova M Kratochvilova M Dusilova-Sulkova S Uhrova J Bandur S Malbohan IM Zima T Tesar V Fibroblast growth factor 23 and matrix-metalloproteinases in patients with chronic kidney disease: are they associated with cardiovascular disease? Kidney Blood Press Res 2009 32 276 283 10.1159/000243050 19797911

[B10] Gutiérrez O Mannstadt M Isakova T Rauh-Hain J Tamez H Shah A Smith K Lee H Thadhani R Jüppner H Wolf M Fibroblast growth factor 23 and mortality among patients undergoing hemodialysis N Engl J Med 2008 359 584 592 10.1056/NEJMoa0706130 18687639 PMC2890264

[B11] Isakova T Xie H Yang W Xie D Anderson AH Scialla J Wahl P Gutierrez OM Steigerwalt S He J Schwartz S Lo J Ojo A Sondheimer J Hsu CY Lash J Leonard M Kusek JW Feldman HI Wolf M Fibroblast growth factor 23 and risks of mortality and end-stage renal disease in patients with chronic kidney disease JAMA 2011 305 2432 2439 10.1001/jama.2011.826 21673295 PMC3124770

[B12] Arnlov J Carlsson AC Sundstrom J Ingelsson E Larsson A Lind L Larsson TE Serum FGF23 and risk of cardiovascular events in relation to mineral metabolism and cardiovascular pathology Clin J Am Soc Nephrol 2013 8 781 786 10.2215/CJN.09570912 23335040 PMC3641622

[B13] Faul C Amaral AP Oskouei B Hu MC Sloan A Isakova T Gutierrez OM Aguillon-Prada R Lincoln J Hare JM Mundel P Morales A Scialla J Fischer M Soliman EZ Chen J Go AS Rosas SE Nessel L Townsend RR Feldman HI St John Sutton M Ojo A Gadegbeku C Di Marco GS Reuter S Kentrup D Tiemann K Brand M Hill JA FGF23 induces left ventricular hypertrophy J Clin Invest 2011 121 4393 4408 10.1172/JCI46122 21985788 PMC3204831

[B14] Stubbs JR Quarles LD Fibroblast growth factor 23: uremic toxin or innocent bystander in chronic kidney disease? Nephrol News Issues 2009 23 33 34 36–37 19534362

[B15] Gutierrez O Isakova T Rhee E Shah A Holmes J Collerone G Juppner H Wolf M Fibroblast growth factor-23 mitigates hyperphosphatemia but accentuates calcitriol deficiency in chronic kidney disease J Am Soc Nephrol 2005 16 2205 2215 10.1681/ASN.2005010052 15917335

[B16] Ferrari SL Bonjour JP Rizzoli R Fibroblast growth factor-23 relationship to dietary phosphate and renal phosphate handling in healthy young men J Clin Endocrinol Metab 2005 90 1519 1524 10.1210/jc.2004-1039 15613425

[B17] Kremsdorf RA Hoofnagle AN Kratz M Weigle DS Callahan HS Purnell JQ Horgan AM de Boer IH Kestenbaum BR Effects of a high-protein diet on regulation of phosphorus homeostasis J Clin Endocrinol Metab 2013 98 1207 1213 10.1210/jc.2012-2910 23393178 PMC3590482

[B18] Oliveira RB Cancela AL Graciolli FG Dos Reis LM Draibe SA Cuppari L Carvalho AB Jorgetti V Canziani ME Moyses RM Early control of PTH and FGF23 in normophosphatemic CKD patients: a new target in CKD-MBD therapy? Clin J Am Soc Nephrol 2010 5 286 291 10.2215/CJN.05420709 19965540 PMC2827593

[B19] Gonzalez-Parra E Gonzalez-Casaus ML Galan A Martinez-Calero A Navas V Rodriguez M Ortiz A Lanthanum carbonate reduces FGF23 in chronic kidney disease stage 3 patients Nephrol Dial Transplant 2011 26 2567 2571 10.1093/ndt/gfr144 21436379

[B20] Isakova T Barchi-Chung A Enfield G Smith K Vargas G Houston J Xie H Wahl P Schiavenato E Dosch A Gutierrez OM Diego J Lenz O Contreras G Mendez A Weiner RB Wolf M Effects of dietary phosphate restriction and phosphate binders on FGF23 Levels in CKD Clin J Am Soc Nephrol 2013 8 1009 1018 10.2215/CJN.09250912 23471131 PMC3675851

[B21] Di Iorio B Di Micco L Torraca S Sirico ML Russo L Pota A Mirenghi F Russo D Acute effects of very-low-protein diet on FGF23 levels: a randomized study Clin J Am Soc Nephrol 2012 7 581 587 10.2215/CJN.07640711 22362063

[B22] Shinaberger CS Greenland S Kopple JD Van Wyck D Mehrotra R Kovesdy CP Kalantar-Zadeh K Is controlling phosphorus by decreasing dietary protein intake beneficial or harmful in persons with chronic kidney disease? Am J Clin Nutr 2008 88 1511 1518 10.3945/ajcn.2008.26665 19064510 PMC5500249

[B23] Takahashi Y Tanaka A Nakamura T Fukuwatari T Shibata K Shimada N Ebihara I Koide H Nicotinamide suppresses hyperphosphatemia in hemodialysis patients Kidney Int 2004 65 1099 1104 10.1111/j.1523-1755.2004.00482.x 14871431

[B24] Isakova T Gutierrez OM Smith K Epstein M Keating LK Juppner H Wolf M Pilot study of dietary phosphorus restriction and phosphorus binders to target fibroblast growth factor 23 in patients with chronic kidney disease Nephrol Dial Transplant 2011 26 584 591 10.1093/ndt/gfq419 20631407 PMC3108359

[B25] Levey AS Bosch JP Lewis JB Greene T Rogers N Roth D A more accurate method to estimate glomerular filtration rate from serum creatinine: a new prediction equation. Modification of Diet in Renal Disease Study Group Ann Intern Med 1999 130 461 470 10.7326/0003-4819-130-6-199903160-00002 10075613

[B26] ICH harmonised tripartite guidelines for good clinical practice 1996 http://www.ich.org/fileadmin/Public_Web_Site/ICH_Products/Guidelines/Efficacy/E6_R1/Step4/E6_R1__Guideline.pdf

[B27] Declaration of Helsinki: ethical principles for medical research involving human subjects http://www.wma.net/en/30publications/10policies/b3/index.html.pdf?print-media-type&footer-right=%5Bpage%5D/%5BtoPage%5D 19886379

[B28] Brochner-Mortensen J A simple method for the determination of glomerular filtration rate Scand J Clin Lab Invest 1972 30 271 274 10.3109/00365517209084290 4629674

[B29] Wolf M Forging forward with 10 burning questions on FGF23 in kidney disease J Am Soc Nephrol 2010 21 1427 1435 10.1681/ASN.2009121293 20507943

[B30] Jonsson KB Zahradnik R Larsson T White KE Sugimoto T Imanishi Y Yamamoto T Hampson G Koshiyama H Ljunggren O Oba K Yang IM Miyauchi A Econs MJ Lavigne J Juppner H Fibroblast growth factor 23 in oncogenic osteomalacia and X-linked hypophosphatemia N Engl J Med 2003 348 1656 1663 10.1056/NEJMoa020881 12711740

[B31] Block GA Wheeler DC Persky MS Kestenbaum B Ketteler M Spiegel DM Allison MA Asplin J Smits G Hoofnagle AN Kooienga L Thadhani R Mannstadt M Wolf M Chertow GM Effects of phosphate binders in moderate CKD J Am Soc Nephrol 2012 23 1407 1415 10.1681/ASN.2012030223 22822075 PMC3402292

[B32] Yilmaz MI Sonmez A Saglam M Yaman H Kilic S Eyileten T Caglar K Oguz Y Vural A Yenicesu M Mallamaci F Zoccali C Comparison of calcium acetate and sevelamer on vascular function and fibroblast growth factor 23 in CKD patients: a randomized clinical trial Am J Kidney Dis 2012 59 177 185 10.1053/j.ajkd.2011.11.007 22137672

