# Supplementary material for: Changes in fibroblast growth factor 23 levels in normophosphatemic patients with chronic kidney disease stage 3 treated with lanthanum carbonate: results of the PREFECT study, a phase 2a, double blind, randomized, placebo-controlled trial
Source: BMC Nephrol. 2014 May 5;15:71. doi: 10.1186/1471-2369-15-71 (PMC4107721; doi:10.1186/1471-2369-15-71)
Supplement: Additional file 1: Table S1 — Change from baseline to week 12 in secondary and tertiary variables. [file 1471-2369-15-71-S1.pdf]

Supplementary Table 1. Change from baseline to week 12 in secondary and tertiary variables

	**Lanthanum carbonate (*n* = 17)**		**Placebo (*n* = 12)**		
	**Baseline value, mean (SD)**	**Change from baseline to endpoint (LOCF), mean (SD)**	**Baseline value, mean (SD)**	**Change from baseline to endpoint (LOCF), mean (SD)**	***P*-value**
cFGF23, RU/ml	188.76 (247.09)	−24.16 (43.57)	130.54 (62.39)	–3.11 (40.98)	0.2068
iPTH, pg/ml	69.4 (30.4)	2.1 (12.1)	75.8 (43.9)	−5.5 (22.6)	0.2995
1,25-dihydroxyvitamin D, pg/ml	43.2 (1709)	0.1 (12.2)	51.1 (11.4)	−9.5 (19.6)	0.3252
Serum phosphate, mmol/l	1.01 (0.18)	0.01 (0.18)	1.05 (0.13)	0.03 (0.12)	0.6134
Serum total calcium, mmol/l	2.36 (0.14)	0.03 (0.17)	2.38 (0.10)	–0.01 (0.13)	0.5636
Serum calcium × phosphate product, $mmol^2/l^2$	2.37 (0.43)	0.07 (0.35)	2.50 (0.30)	0.06 (0.33)	0.9220
Urinary fractional phosphate	27.0 (10.1)	–6.8 (6.8)	24.2 (12.3)	–0.7 (5.7)	0.1459

excretion, %					
24-h urinary calcium excretion, mmol[a]	1.87 (0.77)	–0.10 (0.72)	1.43 (0.66)	0.96 (1.29)	0.0371
24-h urinary phosphate excretion, mmol[b]	18.2 (6.8)	–3.7 (8.3)	16.2 (9.1)	4.0 (5.1)	0.0162
Urine creatinine, mmol/l[b]	6.26 (3.54)	–0.22 (1.75)	5.61 (1.95)	–0.33 (1.39)	0.6688
Urine urea nitrogen concentration, mmol/l[b]	163.0 (63.8)	7.8 (41.0)	182.1 (83.8)	–10.1 (57.8)	0.5127
25-hydroxyvitamin D, ng/ml	39.9 (14.1)	–10.1 (6.5)	40.2 (7.0)	–11.3 (9.8)	0.6649
Ionized calcium, mmol/l[c]	1.212 (0.044)	0.012 (0.049)	1.220 (0.029)	–0.004 (0.028)	0.4283
CrossLaps®, pmol/l	3833.5 (2083.1)	347.4 (821.2)	3761.9 (2087.9)	178.9 (867.8)	0.5793
Bone-specific alkaline phosphatase, ng/ml	12.1 (3.2)	0.8 (2.0)	11.4 (3.7)	0.0 (2.4)	0.2613
Serum creatinine, mmol/l	158.9 (48.4)	−1.9 (16.1)	127.1 (42.9)	–3.7 (12.6)	0.2879
iGFR, ml/min/1.73 m^2	42.2 (10.1)	−0.2 (4.9)	48.0 (13.1)	–0.2 (10.4)	0.5105

P-values from ANCOVA models using change from baseline of least-squares mean values at week 12 with treatment group as a factor and the corresponding value at baseline as a covariate.

[a]Two patients missing from the placebo group and one from the lanthanum carbonate group at baseline and endpoint.

[b]One patient missing from the placebo group at baseline and endpoint.

[c]One patient missing from the placebo group at baseline and two at endpoint.

ANCOVA, analysis of covariance; cFGF23, C-terminal fibroblast growth factor 23; iGFR, iohexol glomerular filtration rate; iPTH, intact parathyroid hormone; LOCF, last observation carried forward; SD, standard deviation.
