# Supplementary material for: Changes in fibroblast growth factor 23 levels in normophosphatemic patients with chronic kidney disease stage 3 treated with lanthanum carbonate: results of the PREFECT study, a phase 2a, double blind, randomized, placebo-controlled trial
Source: BMC Nephrol. 2014 May 5;15:71. doi: 10.1186/1471-2369-15-71 (PMC4107721; doi:10.1186/1471-2369-15-71)
Supplement: Additional file 2: Figure S1 — Change in (a) intact parathyroid hormone and (b) 1,25-dihydroxyvitamin D throughout the study. [file 1471-2369-15-71-S2.pdf]

Supplementary Figure 1. Change in (a) iPTH and (b) 1,25-dihydroxyvitamin D over the course of the study.

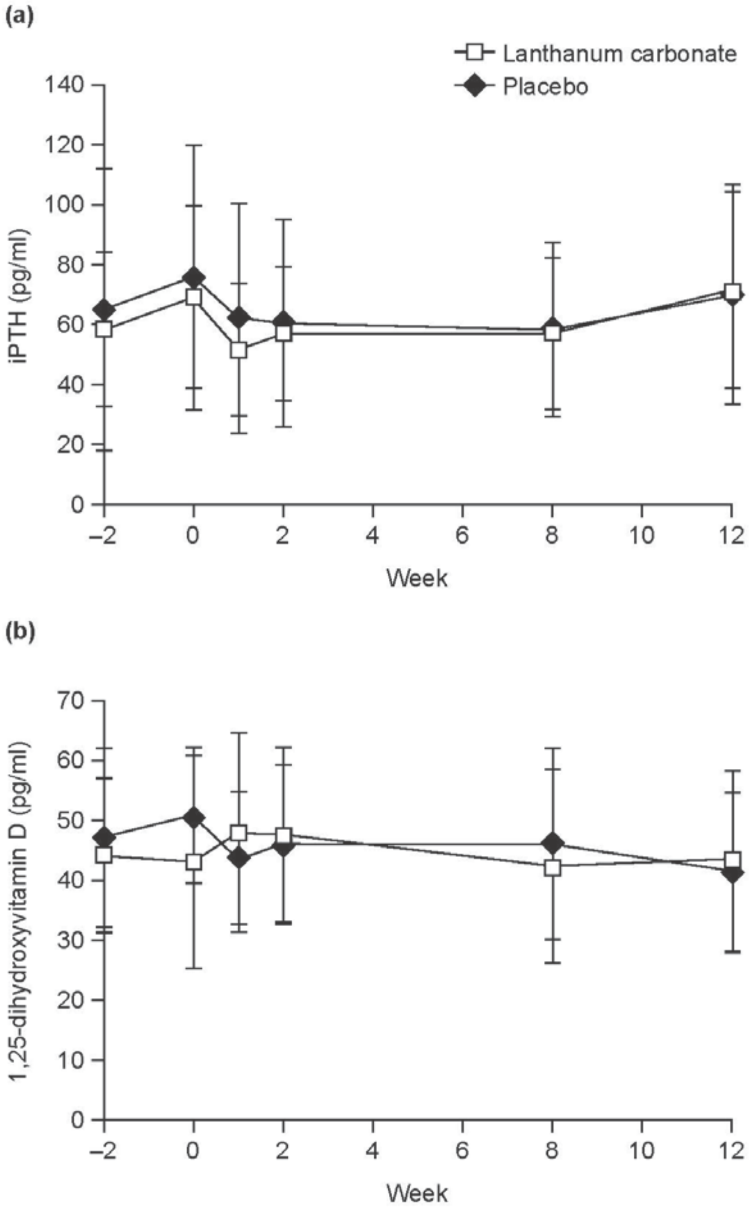

Graphs show mean ± standard deviation.

iPTH, intact parathyroid hormone

Lanthanum carbonate, *n* = 17; placebo *n* = 12.
