# Supplementary material for: Changes in fibroblast growth factor 23 levels in normophosphatemic patients with chronic kidney disease stage 3 treated with lanthanum carbonate: results of the PREFECT study, a phase 2a, double blind, randomized, placebo-controlled trial
Source: BMC Nephrol. 2014 May 5;15:71. doi: 10.1186/1471-2369-15-71 (PMC4107721; doi:10.1186/1471-2369-15-71)
Supplement: Additional file 3: Figure S2 — Post hoc analysis of iFGF23 levels. Graphs show LS mean percentage change from baseline of iFGF23 by treatment and by baseline levels of (a) estimated glomerular filtration rate (eGFR), (b) C-terminal FGF23 (cFGF23), (c) intact parathyroid hormone (iPTH) and by (d) estimated daily phosphate intake and (e) gender. [file 1471-2369-15-71-S3.pdf]

Supplementary Figure 2. ***Post hoc*** **analysis of iFGF23 levels. Graphs show LS mean percentage change from baseline of iFGF23 by treatment and by baseline levels of (a) eGFR, (b) cFGF23, (c) iPTH and by (d) estimated daily phosphate intake and (e) gender.**

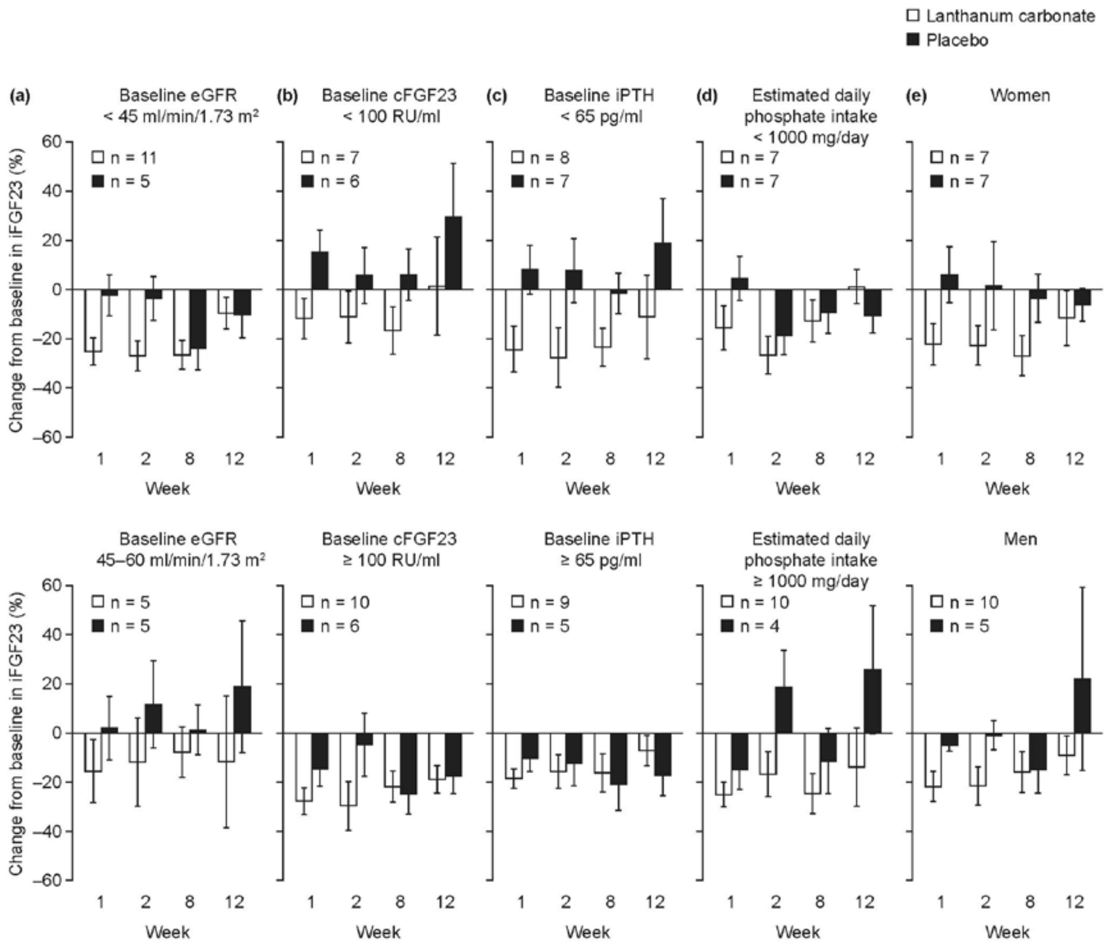

Graphs show percentage change in LS mean ± standard error.

cFGF23, C-terminal fibroblast growth factor; eGFR, estimated glomerular filtration rate; iFGF23, intact fibroblast growth factor 23; iPTH, intact parathyroid hormone; LS, least-squares.
